# Special Issue: Fungal–Bacterial Interactions—Current Knowledge and Future Perspectives

**DOI:** 10.3390/jof5040089

**Published:** 2019-09-24

**Authors:** Paul L. Fidel, Mairi C. Noverr

**Affiliations:** 1 Center of Excellence in Oral and Craniofacial Biology, School of Dentistry, Louisiana State University Health Sciences Center, New Orleans, LA 70119, USA; 2 Department of Microbiology and Immunology, School of Medicine, Tulane University, New Orleans, LA 70112, USA

We would like to thank all the contributors to the Special Issue on Fungal–Bacterial Interactions—Current Knowledge and Future Perspectives. In total, seven (7) reviews/perspective papers were published in the issue. A wide range of various fungal–bacterial interactions were covered. Two papers covered *Candida albicans* and *Staphylococcus aureus* interactions, one from the *Candida* perspective (Esher et al.) [1], and the other from the *Staphylococcus* perspective (Todd and Peters) [2]. Another paper detailed the interactions between *Aspergillus fumigatus* and *Pseudomonas aeruginosa* (Briard et al.) [3]. Other studies included the interactions between *Candida albicans* and *Pseudomonas aeruginosa* (Fourie and Pohl) [4], bacterial interactions of *Cryptococcus neoformans* (Mayer and Kronstad) [5], and dysbiotic interactions between *Candida albicans* and the oral bacterial microbiome that contribute to the pathogenesis of oral candidiasis (Bertolini and Dongari-Bagtzoglou) [6]. Lastly, another paper covered the new emerging powerful antifungal action of the Type VI secretion system (T6SS) of many Gram-negative bacteria (Trunk et al.) against several fungal species [7]. All the papers provide a wealth of information about what is currently known on the various fungal–bacterial interactions, as well as highly insightful future perspectives, often with provocative hypotheses that will direct future research in each field of study. Again, we wish to thank all the authors and the reviewers for their significant contributions to this Special Issue and for making it a highly successful and timely collection of studies.
